# Analysis of a Combination Therapy Protocol for the Treatment of Oral Mucous Membrane Pemphigoid: A Retrospective Case Series Study

**DOI:** 10.1155/2024/5524514

**Published:** 2024-02-08

**Authors:** Simona Santonocito, Alessandro Polizzi, Marco Matarese, Rosario Caltabiano, Gaetano Isola

**Affiliations:** ^1^Department of General Surgery and Surgical-Medical Specialties, School of Dentistry, University of Catania, Via S. Sofia 78, Catania 95124, Italy; ^2^Department of Biomedical and Dental Sciences and Morphofunctional Imaging, University of Messina, Via Consolare Valeria 1, Messina 98123, Italy

## Abstract

Mucous membrane pemphigoid (MMP) is an autoimmune-based bullous disease affecting the mucous membranes, mainly oral and ocular. One of the most common clinical manifestations is desquamative gingivitis (DG), characterized by intense symptoms and functional limitations. The dentist is among the first specialists to observe DG and, therefore, must be able to diagnose it. In this regard, the purpose of the present study was to evaluate the efficacy and safety of a clinical protocol for the topical management of patients with DG and MMP buccal lesions. Thirteen patients with clinical and histologic diagnoses of MMP-localized DG in the oral cavity were retrospectively enrolled. Each patient received topical treatment with clobetasol propionate oral gel 0.05%; nicotinamide; oral probiotic (contains *Bifidobacterium lactis HN019*, *Kluyveromyces marxianus fragilis B0399*, colostrum, and biotin); and doxycycline. Before and after 3 months of therapy, clinic records were collected for each patient. Seven patients (53.8%) had a complete response to treatment; four patients (30.8%) had a partial response to treatment; and, finally, two patients (15.4%) had no benefit from therapy. Dental management of patients presenting solely with oral manifestations of MMP may involve the use of topical corticosteroids, doxycycline, vitamin supplements, and probiotics and associating professional oral hygiene procedures.

## 1. Introduction

Mucous membrane pemphigoid (MMP) comprises a group of subepithelial, chronic, progressive vesiculobullous diseases of autoimmune etiology that predominantly affect the mucous membranes, oral and ocular and, rarely, the skin [1, 2]. The immune system produces autoantibodies directed against different components of the basement membrane zone (BMZ), including BP180, BP230, Laminin 332, Type VII collagen, Integrin *α*6*β*4, LM*γ*1, and other as yet unknown autoantigens, inducing linear deposition of IgG and/or IgA in the BMZ [3]. When the lesions involve only the oral mucosa and/or skin, MMP is termed “low risk”; sometimes, similar to lichen planus lesions [4, 5], are involved the ocular, esophageal, laryngeal, nasopharyngeal, and anogenital mucosa, it is “high risk.” The lesions, especially extraoral lesions, tend to create scarring on the mucous membranes and for this reason, MMP was earlier indicated as “scarring pemphigoid.” Scarring outcomes can cause major sequelae, such as reduced visual acuity, blindness, and supraglottic stenosis with hoarseness or airway obstruction [3]. A greater predilection for the female sex has been observed with 1.5–2 times more frequently than men, without racial or geographic preference [6]. However, in a study conducted in northern Israel in the years 2000–2015, it was observed that the incidence rate of bullous pemphigoid (BP) is significantly higher among Jews than among Arabs. This result indicate that some ethnic groups may have an ethnic predisposition to the development of BP to be attributed, probably, to the existence of a genetic component in the pathogenesis of the disease [7]. European data show an incidence of about 1 : 1,000,000 per year [8]. However, over the past few decades, there has been a slight increase in cases as a result of an aging population [2]. The oral cavity is the most frequently affected site, mainly affecting the gums and presenting clinically in the form of desquamative gingivitis (DG), which clinically presents as an erythematous band along the gingival tissue accompanied by dryness and scaling [9]. DG may represent the clinical sign of several mucocutaneous disorders, including oral lichen planus (OLP), pemphigoid, and pemphigus [10] and, to a lesser extent, other immunological disorders such as lupus erythematosus, erythema multiforme, graft versus host disease, epidermolysis bullosa acquisita, plasma cell gingivitis (PCG), and collagen diseases [11]. Due to its autoimmune nature, gingivitis in MMP does not improve with the elimination of plaque and calculus, unlike common periodontal disease induced by the accumulation of a dysbiotic biofilm on the surface of the dental elements. In 90% of cases, MMP can also affect the buccal mucosa, soft palate, and lips. Oral blisters tend to burst due to the insults they are subjected in chewing, phonation, and swallowing, leaving as an outcome painful erosions and ulcers that tend to heal slowly [12]. Diagnosis is based on a combination of clinical and histologic aspects [13]. Histologic examination shows detachment of the epithelium from the underlying basement membrane with an inflammatory infiltrate consisting of eosinophils, lymphocytes, and neutrophils. Direct immunofluorescence reveals linear deposition of IgG, C3, and sometimes IgA along the basement membrane. Direct immunofluorescence is useful in the differential diagnosis with pemphigus vulgaris (P.V.), OLP, and systemic lupus erythematosus, which may present similar histologic pictures [14]. The clinical management of patients with MMP is very complex and involves the use of both systemic and topical corticosteroids. The discriminant between the two treatment modalities is the severity of the disease. In severe forms with ocular, genital, nasopharyngeal, esophageal, or laryngeal involvement, systemic corticosteroids are involved. In less severe forms with localized, oral, or cutaneous involvement, the first-line treatment is the application of topical corticosteroids to the affected areas [12, 15, 16]. Several guidelines have attempted to standardize the management of MMP and other autoimmune-based bullous diseases but with little success. Clinical management remains controversial and complex, especially for the dental professional, who is often called upon to manage this class of patients.

The purpose of treating MMP is primarily disease control, defined as the stage at which new inflammatory lesions cease to form and established lesions begin to heal. Immunosuppressive agents represent the first-line drugs for the treatment of MMP and should be chosen with a “stepwise” approach, starting with the drugs that induce the fewest side effects. Therefore, when possible, topical therapies should be preferred. Currently, topical therapies for the treatment of oral MMP include corticosteroids, such as oral clobetasol, which have been shown to be extremely effective in reducing symptoms [17]. However, topical corticosteroids do not affect the immune-mediated course of the disease, unlike other drugs, including systemic corticosteroids and tetracyclines. In addition to their ability to control bacterial proliferation and growth, the latter possess anti-inflammatory and collagenolytic properties. Because of these important properties, tetracyclines have been proposed as a first-line agent in mild/moderate MMP, partly because of its reduced number of side effects compared with corticosteroids and other conventional immunosuppressive agents. Based on this observation, a combined approach in which the benefits of topical therapies are combined with those of systemic therapies may be useful, because acting on different pathogenetic mechanisms, they could promote better disease control [17].

In light of the above, the purpose of the present study was to evaluate the efficacy and safety of a combination clinical protocol for the management of patients with DG and MMP buccal lesions.

## 2. Materials and Methods

### 2.1. Study Design and Population

Thirteen patients with clinical and histological diagnoses of MMP-localized gingivitis in the oral cavity and treated at the dental clinic of Policlinic G. Rodolico in Catania, Italy, were retrospectively enrolled between March 2018 and December 2022. The methods of selecting patients included in the following case series were: clinical and histological diagnosis of pemphigoid, absence of extraoral bullous lesions, and absence of previous therapy in the previous 6 months. Patients without follow-up, patients with other autoimmune-based diseases, patients on medications that can induce bullous lesions, and patients with cognitive impairment were excluded from the study. Voluntary consent to participate was obtained for all patients included in the study, and each was informed about possible adverse effects of therapy.

### 2.2. Protocol of Treatment and Field Data Collection

For each patient included in the study, demographic, clinic-pathological, and treatment data were recorded from medical records. Each patient received topical treatment with clobetasol propionate oral gel 0.05%, in adhesive base, to be applied twice daily for 90 days on the lesion in combination with nicotinamide 500 mg twice daily for 90 days; oral probiotic (contains *Bifidobacterium lactis HN019*, *Kluyveromyces marxianus fragilis B0399*, colostrum, and biotin) one tablet a day for 90 days; doxycycline 100 mg two tablets daily for 5 days. None of the patients had previous topical or systemic drug therapy in the previous 6 months. The patients were instructed about the proper way to apply the topical gel, namely to avoid ingestion of the gel and not to drink or eat for at least 40 min after application. Before the start of therapy, patients underwent professional oral hygiene procedures to reduce bacterial biofilm. Complete clinical response was considered when patients stopped developing new lesions during therapy and topical clobetasol tapering could be carried out until minimal dosage or discontinuation without new lesions. Partial clinical response was considered when patients stopped developing new lesions during treatment, but the daily frequency of topical corticosteroid application could not be reduced because of the development of new lesions after dose reduction. An absence of clinical response was considered when patients continued developing new lesions despite topical clobetasol treatment. The following classification system for therapy response is based on that used in the work of Gual et al. [18]. All patients, both at baseline and after 3 months of therapy, underwent photographic examination and a thorough oral and periodontal tissue examination, in which lesion area and lesion number were noted.

## 3. Results

Thirteen patients, including nine females and four males, with localized MMP in the gums and oral mucosa were enrolled in the present study. All patients discontinued the oral probiotic and nicotinamide after 3 months of therapy. After 3 months of therapy, seven patients (53.8%) had a complete response to treatment and underwent a gradual reduction in the frequency of daily topical corticosteroid application until it was discontinued after 4 weeks. Four patients (30.8%) had a partial response to treatment after 3 months of therapy, so daily applications of topical corticosteroid were reduced to the lowest daily dose that could lead to disease control. Finally, two patients (15.4%) had no benefit from therapy and had to discontinue treatment early because of the appearance of lesions in extraoral sites that required switching to systemic corticosteroid therapy. Of the 13 patients observed, only one patient developed side effects during treatment. This patient developed a fungal infection that returned after therapy with the oral suspension of nystatin 1 : 100,000, taken four times daily until the fungal pseudomembranes disappeared (Table 1).

**Figure 1:**
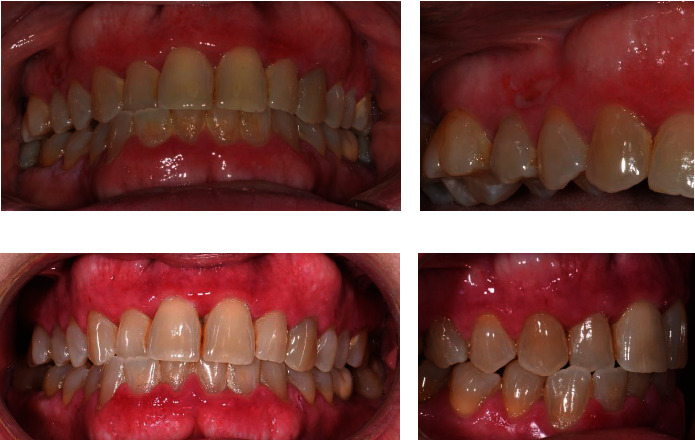
(a) Pretherapy intraoral photos. (b) Subepidermal bullous lesion detail at the keratinized gingiva. (c, d) Intraoral photos after 3 months of therapy show complete control of gingival inflammation and good control of bullous lesions.

**Figure 2:**
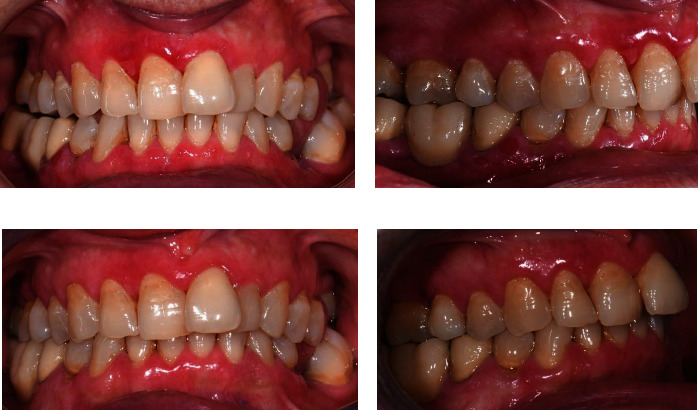
(a) Baseline intraoral photos. (b) Subepidermal bullous lesion detail at the keratinized gingiva. (c, d) Intraoral photos were found after 3 months of therapy, which has partial control of gingival inflammation and discrete control of bullous lesions.

Figures 1 and 2 show the pretherapy and posttherapy intraoral photos of two patients enrolled in the present study.

## 4. Discussion

The analysis of the following clinical cases shows that the combined treatment protocol, which acts on different pathogenic pathways, allows good clinical control of oral MMP and DG forms. In detail, topical application of corticosteroids would enable good control of symptoms, doxycycline and nicotinamide would allow for good control of systemic inflammation, and finally, probiotics would provide for balancing oral dysbiosis. The management of MMP is very complicated and closely related to its severity, which depends on several factors, including the propensity to generate scarring and response to therapy. In detail, the forms that affect only the oral mucosa or skin are definable as “low risk” and easier to manage clinically. In contrast, individuals in whom the ocular, esophageal, laryngeal, nasopharyngeal, and anogenital mucosae are affected, which are defined as “high risk,” have complex clinical management, involving multidisciplinary involvement of multiple specialists such as otolaryngologists, dermatologists, rheumatologists, ophthalmologists, etc [5].

In the present cases, the patients present a low-risk form with only oral mucosal involvement, with the site of choice being the gingiva. Gingival lesions, such as in our cases, could be misinterpreted as periodontitis; therefore, a scrupulous differential diagnosis with mucosal pemphigoid, BP, pemphigus vulgaris, and bullous lichen planus must be made. Patients with MMP should undergo meticulous dental inspection as they are more susceptible to developing periodontal disease [19]. Several studies have indicated that subjects with MMP-induced DG have worse gingival–periodontal status than controls, with deeper pockets and greater loss of clinical attachment level [11, 20]. Patients with oral MMP have difficulty maintaining proper oral hygiene, as home hygiene maneuvres trigger pain and gingival bleeding. Therefore, gingival lesions from MMP can promote plaque accumulation and thus promote periodontal disease. The inflammatory process locally associated with periodontitis could trigger and perpetuate the autoimmune response, inducing a more pronounced presentation of antigenic epitopes in the damaged periodontium [19]. In addition, the erosive gingival lesions can serve as a reservoir of microbial plaque and generate an inflammatory response like that observed in DG, including leukocyte infiltration and the release of proinflammatory cytokines and matrix metalloproteinases. Therefore, in patients with MMPs, the inflammatory process associated with periodontal disease may trigger and/or perpetuate an autoimmune response by inducing a poor response of the gingival lesions to immunosuppressive drugs, especially systemic ones [21]. Professional oral hygiene procedures improve gingival status and reduce gum-related pain and the risk of developing periodontitis [14]. Findings from a systematic review conducted by Garcia-Pola et al. [22] show that in patients with DG, the combination of appropriate daily gingival hygiene techniques and the periodic performance, according to particular needs, of periodontal treatment based on scaling and root planing (SRP) decreases pain perception, disease progression, dental plaque, and gingival bleeding, representing one of the main adjutants for the specific treatment of DG. The treatment of choice for MMP involves the use of corticosteroids [17]. Keeping in mind that we are dealing with forms with only oral expression, we chose to intervene through topical corticosteroid therapy based on clobetasol propionate 0%–0.05%. Clobetasol propionate was found to have good efficacy, compared with other less potent topical corticosteroids, in the treatment of oral vesiculosus diseases, including MMP, especially in the recalcitrant forms of the disease [23]. In particular, it was observed to induce faster pain control (within 7 days) than fluocinonide [24]. This difference may be explained by both the different potency of the two drugs and the greater vasoconstricting power of clobetasol compared with fluocinonide. In detail, clobetasol, by reducing the local immunologic response and causing vasoconstriction to a greater extent than fluocinonide, would promote better control of symptomatology [24]. Doxycycline has shown good results, as already reported in the literature, for the treatment of autoimmune vesiculobullous diseases, thanks to its anti-inflammatory, collagenolytic, and immunosuppressive properties [25]. Doxycycline and other tetracyclines have been proposed as first-line drug in the treatment of mild MMP as they have fewer long-term side effects compared to prolonged therapy with systemic corticosteroids. Our protocol, which showed good results, included a limited time of doxycycline administration as major side effects were observed in prolonged use [26]. However, other evidence suggests prolonged use of tetracycline, between 6 and 8 weeks, with equally good results [27]. Current evidence suggests that combined therapy between superpotent topical corticosteroids and oral doxycycline allows good disease control, representing an alternative therapy to long-term systemic corticosteroids [27]. Numerous evidence has suggested combining tetracycline therapy with nicotinamide [28] or vitamin B3. The exact mechanism by which nicotinamide acts in autoimmune blistering disorders is unclear. However, nicotinamide has known anti-inflammatory properties, with inhibition of proinflammatory cytokines such as interleukin 1*β*, interleukin 6, interleukin 8, and tumor necrosis factor [25]. The use of oral probiotics in the management of autoimmune diseases has gained considerable importance in recent years following observations of how human microbiome might be involved in the development of autoimmunity. Loss of immune tolerance may be caused by changes in microbial composition, which could induce an immune response against the host [29]. In a recent pilot study, it was observed that in subjects with pemphigus and BP, there is an imbalance in the oral microbiota, characterized by a reduction in Bacteroidetes and an increase in the phylum Firmicutes and the genus *Staphylococcus* [30]. Based on this evidence are new and promising therapeutic approaches, such as probiotics or prebiotics, especially in the adjuvant and prolonged treatment of MMP [31]. To rebalance the oral microbiota, oral probiotics could be important adjuvant factors to first-line therapies in the long-term management of MMP patients [32]. Fungal infections of the oral mucosa are known to be one of the most common side effects of prolonged use of topical corticosteroids [26]. Only one patient developed fungal overinfection as an adverse event to the protocol used, but it should be emphasized that he was a subject with a previous history of carcinoma and a compromised immune system from previous cancer therapies. In cases of nonresponse to topical steroids, topical use of the immunomodulator tacrolimus (Ointment Protopic 0.1%) is possible [33]. Patients with multiple, resistant oral lesions and involvement of other mucous membranes should be referred to a dermatologist or internist for systemic therapy [34]. The lack of data on the efficacy of drugs on pain and lesion size is a limitation of the present study. Therefore, further clinical trials evaluating the response of the following protocol on pain and lesion size would be recommended in the future.

## 5. Conclusion

Dentists are the first health professionals to identify this condition. Gingival lesions in patients with MMP are usually treated with improved oral hygiene and topical corticosteroid therapy, to which antibiotic therapy may be useful in the early stages. Therefore, in the future, the possibility of introducing prebiotics and probiotics into the clinical routine to rebalance the oral microbiota and limit the use of antibiotic therapies may prove beneficial. Finally, the collaboration of the dentist with a multidisciplinary medical team that includes an internist, a dermatologist, and an ophthalmologist is essential for managing any resistance to topical treatment or for preventing and managing any extraoral complications that could arise.

## Figures and Tables

**Table 1:** Demographic and medical history data and clinical response to treatment of the sample studied.

Gender	Age at diagnosis (years)	Comorbidities	Number of lesions before therapy	Number of lesions after therapy	Clinical response	Adverse effects	Suspension
F	54	None	4	0	Complete	—	—
F	78	Hypertension, dyslipidemia, and breast cancer	5	2	Partial	Candida overinfection	—
M	38	None	6	0	Complete	None	—
F	81	Hypertension, dyslipidemia, diabetes, and stroke	5	8	Absent	None	Occurrence of esophageal and laryngeal
F	57	Depression	5	0	Complete	None	—
M	48	Hypertension	3	0	Complete	None	—
F	64	Hypertension and depression	3	1	Partial	None	—
F	79	COPD ^*∗*^	7	3	Partial	None	—
F	51	Asthma	3	0	Complete	None	—
M	74	Hypertension and prostate cancer	3	0	Complete	None	—
M	76	Coronary heart disease	5	2	Partial	None	—
F	67	Diabetes	4	0	Complete	None	—
F	61	Rheumatic disease	5	6	Absent	None	Occurrence of ocular lesions

^*∗*^Chronic obstructive pulmonary disease.

## Data Availability

The data used to support the findings of this study are freely available from the corresponding author on reasonable request.
